# Organ donation in Germany: opt-in vs. opt-out—opinions and voting patterns in the 19th German Bundestag

**DOI:** 10.3389/frtra.2025.1526238

**Published:** 2025-02-27

**Authors:** Asli Zeybek, Nguyen-Son Le, Utz Settmacher, Martina Koch, Helmut Arbogast, Svitlana Ziganshyna, Klaus Hahnenkamp, Björn Nashan

**Affiliations:** ^1^ Department of Internal Medicine, Zuger Kantonsspital, Baar, Switzerland; ^2^ Department of Internal Medicine 2, University Hospital Krems, Karl Landsteiner Private University for Health Sciences, Krems an der Donau, Austria; ^3^ Department of General, Visceral and Vascular Surgery, University Hospital Jena, Jena, Germany; ^4^ Department of General, Visceral and Transplant Surgery, University Hospital Mainz, Mainz, Germany; ^5^ Department of General, Visceral and Transplant Surgery, LMU-Clinic Munich – Campus Großhadern, München, Germany; ^6^ Organ Donation Coordinator Unit, University of Leipzig Medical Center, Leipzig, Germany; ^7^ Department of Anaesthesia, Intensive Care, Emergency and Pain Medicine, University Clinic Greifswald, Greifswald, Germany; ^8^ Department of Hepato-Pancreatico-Biliary Surgery and the Transplantation Center, University of Sciences & Technology of China, Anhui, China

**Keywords:** organ donation, German Bundestag, opt-out, transplant legislation, voting behavior, Germany

## Abstract

In early 2024, 8,394 patients were waitlisted for solid organ transplantation in Germany. Long waiting times and declining donor numbers highlight the urgency for political measures to improve the organ donation system. This retrospective analysis examined the attitudes of the 19th German Bundestag members towards organ donation and their voting behavior on the opt-out system, which was decided on January 6, 2020. The results were analyzed in relation to party affiliation, age, gender, and educational background. Among members of parliament (MP), 34% were in favor for organ donation, 8% were critical, and 58% made no statement on organ donation at all. Younger members were less likely to express an opinion than older ones (*p* < 0.001). CDU/CSU (50%) and members of the SPD (48%) showed the highest approval, while The Green Party (39%) showed the lowest approval rate. AfD members had the highest abstention rate (96%, *p* < 0.001). SPD (66%, OR 33.24) and CDU/CSU (63%, OR 28.32) strongly supported the opt-out system, while AfD (94%) and The Green Party (88%) strongly rejected. Overwhelming majorities of the AfD (94%), The Green Party (89%), and FDP (81%) members who had not previously expressed an opinion to organ donation and legislation voted against the opt-out system, whereas majorities of SPD (66%) and CDU/CSU (66%) voted in favor. Most members who held opposing views on organ donation voted against the opt-out solution. Party affiliation was strongly correlated with both attitudes towards organ donation and voting behavior as well as a considerable lack of in-depth knowledge regarding transplant legislation. A fact-based discussion involving medical professionals, who play a key role in the organ donation process, is essential, along with a thorough understanding of the organ transplant law.

## Introduction

In early 2024, 8,394 patients were waitlisted in Germany for solid organ transplantation, with kidneys as leading organ (1). The average wait for a donor kidney is 8–9 years, with significant regional differences (2). After a decline in donors since 2018, numbers returned to previous levels in 2023 (3). The 2020 “Law to Strengthen Decision-Making in Organ Donation” introduced an online register to increase donations, but a proposed opt-out law, failed to pass (4).

Germany's transplantation law mandates explicit consent (*Entscheidungslösung*) for post-mortem organ donation [Transplantation Law, §3, (1), 1] (5), encouraging individuals to document their decision via an online registry or donor card. In case a documentation cannot be found § 4 stipulates, that the next relatives have to be asked for the presumed consent of the deceased. Organ donation without a documented will or approval by the next relatives is not allowed. The law also prohibits donation after circulatory death (DCD) and highlights the special status of living donation, with strict safeguards for living donors to protect their rights and health. However, many citizens remain undecided, with undocumented consent and family opposition being major barriers to donation (6). Public misrepresentation of the proposed opt-out system (7), which would not touch the need to counsel the next relatives for approval (§ 4, paragraph 3, 4)—often framed as “organs can be taken without consent”—has fueled resistance, raising concerns about individual freedom of choice.

This study explores the attitudes of MP of the German Bundestag on organ donation, as their stance influences the country's organ donation policies.

## Materials and methods

This retrospective study investigated the attitudes of MPs of the 19th German Bundestag (as of March 25, 2018) towards organ donation, which were identified through an internet search using their names along with the term “organ donation.” These attitudes were assessed using various sources. First, their support or opposition to specific legislative proposals related to organ donation was considered as an indicator of their attitude. Additionally, statements made in parliamentary debates, interviews at events, and public speeches on the topic were analyzed.

Attitudes were classified as “positive”, “negative”, or “no statement”. Positive views supported organ donation and its political relevance, while negative views expressed skepticism, distrust, or ethical concerns. Correlation between these attitudes and members' gender, age, party affiliation, and education were analyzed.

The second part analyzed the voting results on the opt-out system from January 16, 2020, excluding members who didn't vote or abstained. Voting behavior was analyzed by party affiliation, gender, age and education. The results were then compared with their prior attitudes. Members who left office before the vote were excluded to ensure comparability. The statistical analyses were performed using SPSS. Logistic regression (univariate and multivariate), Chi-square, and Fisheŕs Exact Test were employed to assess the relationship between Bundestag memberś attitude or voting behaviour and variables such as gender, age, party affiliation, and education. Significant variables from univariate analyses were included in multivariate analyses. A Forest Plot was created to display odds ratios from the logistic regression. *P*-values ≤0.05 were considered statistically significant.

## Results

The 19th German Bundestag, elected on September 24, 2017, consisted of 709 MP from CDU/CSU, SPD, AfD, FDP, Linke, The Green Party, and two independents. CDU/CSU had the highest number of seats (246), followed by SPD (153), AfD (92), FDP (80), Linke (69), and The Green Party (67).

MP's age ranged from 28 to 80, with a median of 53 years. Generation cohorts were: 23% born between 1940 and 1959, 64% between 1960 and 1979, and 14% between 1980 and 1992. The Bundestag consisted of 31% female and 69% male (Table 1A).

**Table 1A:** Overview of the study cohort.

Study cohort	Number of members of parliament (%)
Member of parliament	693 (98%)
Resigned members of parliament	16 (2%)
Age (years, median, range)	53.0 (28–80)
Age group	
1940–1959	159 (23%)
1960–1979	441 (64%)
1980–1992	93 (14%)
Gender	
Male	481 (69%)
Female	212 (31%)
Party	
CDU/CSU	241 (35%)
Linke	69 (10%)
FDP	77 (11%)
The Green Party	65 (9%)
SPD	147 (21%)
AfD	92 (13%)
Non-affiliated	2 (0.3%)
School graduation	693 (100.0%)
Vocational training	
Yes	172 (25%)
No	521 (75%)
Higher education degree	
Yes	576 (83%)
No	117 (17%)
UAS/University	
University of Applied Sciences	67 (12%)
University	509 (88%)
University degree	
Social sciences	223 (44%)
STEM	58 (11%)
Law	154 (30%)
Medicine	12 (2%)
Teaching degree	38 (8%)
Arts	15 (3%)
Others	8 (2%)
Medicine & law	1 (0.2%)

UAS, University of Applied Sciences; STEM, science, technology, engineering and mathematics.

All MPs had graduated from high school, and 83% held higher education degrees (University of Applied Sciences or University), primarily in social sciences (44%), law (30%), and STEM (science, technology, engineering, mathematics) (11%).

### Attitude of members of parliament towards organ donation

The study revealed that 58% of the MPs had not made any statement on organ donation at all. Of the 42% who did, 34% were in favor and 8% negative. Younger parliament members (MPs) (born 1980–1992) were less likely to comment, while older MPs were more likely to express an opinion (*p* < 0.001).

Among female MPs, 37% supported and 13% opposed organ donation, compared to 33% and 6% of male MPs, respectively (*p* = 0.004). Positive views were most among CDU/CSU (50%) and SPD (48%), while AfD and FDP had the highest abstention rates (*p* < 0.001).

Educational background had minor influence, but university graduates were more likely to support organ donation compared to those from applied sciences (*p* = 0.043), with field of study also playing a role (*p* < 0.001) (Table 1B).

**Table 1B:** Association between study variables and the attitude of the members of the 19th German Bundestag towards organ donation.

	Attitude towards organ donation			*p*-value
	Negative	Positive	No statement	
Cohort				
1940–1959	16 (10%)	67 (42%)	76 (48%)	**<0.001** *
1960–1979	36 (8%)	156 (35%)	249 (57%)	
1980–1992	5 (5%)	14 (15%)	74 (80%)	
Gender				
Male	30 (6%)	159 (33%)	292 (61%)	**0.004** **
Female	27 (13%)	78 (37%)	107 (51%)	
Party				
CDU/CSU	7 (3%)	120 (50%)	114 (47%)	**<0.001** *
Linke	17 (25%)	11 (16%)	41 (59%)	
FDP	4 (5%)	21 (27%)	52 (68%)	
The Green Party	25 (39%)	12 (19%)	28 (43%)	
SPD	2 (1%)	70 (48%)	75 (51%)	
AfD	2 (2%)	2 (2%)	88 (96%)	
Non-affiliated	0 (0%)	1 (50%)	1 (50%)	
Vocational training				
Yes	16 (9%)	52 (30%)	104 (61%)	0.426**
No	41 (8%)	185 (36%)	295 (57%)	
Higher education degree				
Yes	46 (8%)	199 (35%)	331 (58%)	0.833**
No	11 (9%)	38 (33%)	68 (58%)	
UAS/university				
UAS	6 (9%)	14 (21%)	47 (70%)	**0.043** *
University	40 (8%)	185 (36%)	284 (56%)	
University degrees				
Social sciences	20 (9%)	67 (30%)	136 (61%)	**<0.001** *
STEM	5 (9%)	16 (28%)	37 (64%)	
Law	6 (4%)	71 (46%)	77 (50%)	
Medicine	0 (0%)	11 (92%)	1 (8%)	
Teaching degree	5 (13%)	12 (32%)	21 (55%)	
Art	3 (20%)	6 (40%)	6 (40%)	
Others	0 (0%)	2 (25%)	6 (75%)	
Medicine & law	1 (100%)	0 (0%)	0 (0%)	

Significant *p*-values are highlighted in bold.

CI, coincidence interval; UAS, University of Applied Sciences; STEM, science, technology, mathematics and engineering.

* Fisher's Exact Test.

** Chi-square-Test.

### Voting results on the opt-out system

In the vote on the opt-out system, 41% (*n* = 292) of Bundestag members voted “yes” and 59% (*n* = 379) voted “no.” A total of 5% (*n* = 35) did not participate, and 0.4% (*n* = 3) abstained, with these 38 members excluded from further analysis.

Age (*p* = 0.555) and gender (*p* = 0.580) had no significant impact on voting behavior. SPD (66%) and CDU/CSU (63%) were the strongest supporters, while opposition was highest among AfD (94%), The Green Party (88%), FDP (80%), and Linke (61%) (*p* < 0.001).

**Figure 1:**
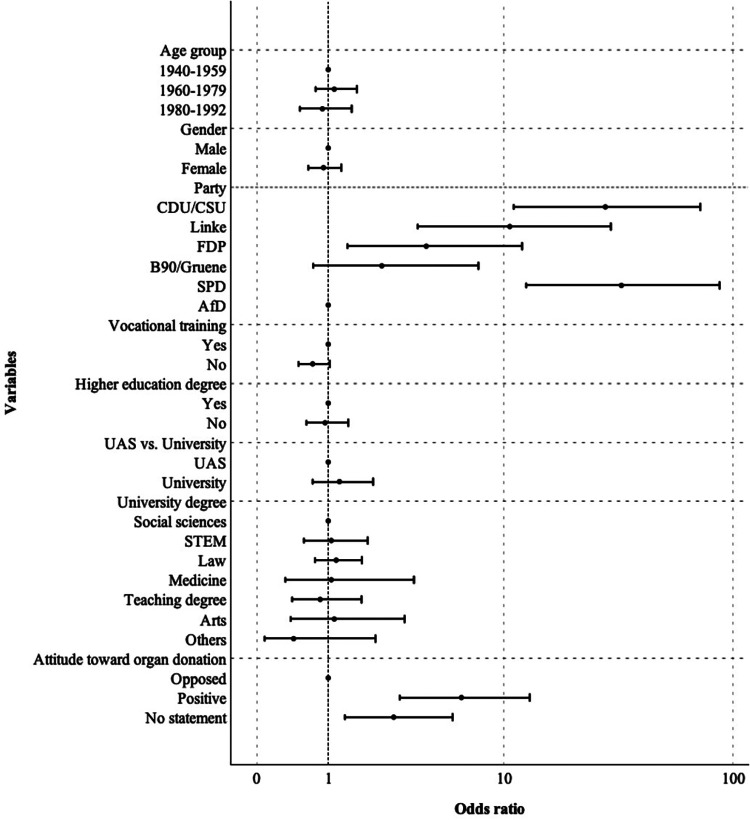
Association between study variables and voting behavior of members of the 19th Bundestag regarding the opt-out system for organ donation. UAS, University of Applied Sciences; STEM, science, technology, engineering, and mathematics.

Educational background had minimal effect, though MPs without vocational training were slightly less likely to vote for the opt-out system (*p* = 0.073). Party affiliation was the main factor influencing voting behavior (*p* < 0.001) (Table 1C and Figure 1).

**Table 1C:** Association between study variables and voting behavior of members of the 19th Bundestag regarding the opt-out system for organ donation.

	Voting behavior		Unadjusted		
	No	Yes	OR	95% CI	*p*-value
Cohort					
1940–1959	84 (58%)	62 (43%)	1.00	Reference	0.555*
1960–1979	229 (55%)	190 (45%)	1.12	0.77–1.64	
1980–1992	55 (60%)	36 (40%)	0.89	0.52–1.51	
Gender					
Male	252 (55%)	203 (45%)	1.00	Reference	0.580*
Female	116 (58%)	85 (42%)	0.91	0.65–1.27	
Party					
CDU/CSU	86 (37%)	145 (63%)	28.32	11.06–72.58	**<0.001** **
Linke	38 (61%)	24 (39%)	10.61	3.76–29.93	
FDP	57 (80%)	14 (20%)	4.17	1.41–12.09	
The Green Party	57 (88%)	8 (12%)	2.36	0.73–7.57	
SPD	46 (34%)	91 (66%)	33.24	12.61–87.62	
AfD	84 (94%)	5 (6%)	1.00	Reference	
Non-affiliated	0 (0%)	1 (100%)	—	—	
Vocational training					
Yes	79 (50%)	79 (50%)	1.00	Reference	0.073*
No	289 (58%)	208 (42%)	0.72	0.50–1.03	
Higher education degree					
Yes	306 (56%)	241 (44%)	1.00	Reference	0.779*
No	62 (57%)	46 (43%)	0.94	0.62–1.43	
UAS/University					
UAS	38 (60%)	25 (40%)	1.00	Reference	0.455*
University	268 (55%)	216 (45%)	1.23	0.72–2.09	
University degree					
Social sciences	120 (56%)	94 (44%)	1.00	Reference	0.843**
STEM	29 (55%)	24 (45%)	1.06	0.58–1.93	
Law	77 (52%)	70 (48%)	1.16	0.76–1.77	
Medicine	6 (55%)	5 (46%)	1.06	0.32–3.59	
Teaching degree	21 (60%)	14 (40%)	0.85	0.41–1.76	
Arts	8 (53%)	7 (47%)	1.12	0.39–3.19	
Others	6 (75%)	2 (25%)	0.43	0.08–2.16	
Medicine & law	1 (100%)	0 (0%)	—	—	
Attitude toward organ donation					
Opposed	44 (82%)	10 (19%)	1.00	Reference	**<0.001** *
Positive	92 (42%)	131 (59%)	6.27	3.00–13.09	
No statement	232 (61%)	146 (39%)	2.77	1.35–5.67	
Multivariant analysis					
Party affiliation	—	—	1.28	1.18–1.40	**<0.001**
Attitude toward organ donation	—	—	0.98	0.77–1.26	0.904

Significant *p*-values are highlighted in bold.

CI, coincidence interval; UAS, University of Applied Sciences; STEM, science, technology, mathematics and engineering.

* Chi-square-Test.

** Fisher's Exact Test.

### Comparison between attitudes and voting results of members of parliament

Analysis revealed that 82% of MPs initially opposed to organ donation voted against the opt-out system, while 18% supported it. Among supporters of organ donation, 59% voted for the opt-out system, and 41% opposed it. MPs with no prior stance mostly voted against the bill (61%, *p* < 0.001).

Most members of The Green Party, SPD and FDP who were initially opposed to organ donation, voted against the opt-out-system, while 43% of CDU/CSU MPs shifted their position. Positive votes were most common among those with an initially positive view, especially in SPD (71%), Linke (70%), and CDU/CSU (61%).

Those without prior opinions who voted against were mainly from AfD (94%), The Green Party (89%), and FDP (81%), while SPD (66%) and CDU/CSU (66%) members mostly supported an opt-out system (Tables 1C, 1D).

**Table 1D:** Analysis of the attitudes and voting behavior of members of the 19th German Bundestag with regard to the opt-out solution for organ donation and the correlation of study variables.

Attitude towards organ donation	Voting		Unadjusted		
	No	Yes	OR	95% CI	*p*-value
Cohorts					
1940–1959					
Negative	12 (80%)	3 (20%)	1.00	Reference	**<0.001** *
Positive	25 (41%)	36 (59%)	5.76	1.47–22.54	
No statement	47 (68%)	22 (32%)	1.87	0.48–7.32	
1960–1979					
Negative	27 (79%)	7 (21%)	1.00	Reference	**<0.001** *
Positive	61 (41%)	88 (59%)	5.56	2.28–13.59	
No statement	141 (60%)	95 (40%)	2.60	1.09–6.21	
1980–1992					
Negative	5 (100%)	0 (0%)	—	—	0.115*
Positive	6 (46%)	7 (54%)	1.77	0.54–5.80	
No statement	44 (60%)	29 (40%)	1.00	Reference	
Gender					
Male					
Negative	23 (77%)	7 (23%)	1.00	Reference	**<0.001** *
Positive	59 (40%)	89 (60%)	4.96	2.00–12.29	
No statement	170 (61%)	107 (39%)	2.07	0.86–4.99	
Female					
Negative	21 (88%)	3 (13%)	1.00	Reference	**<0.001** *
Positive	33 (44%)	42 (56%)	8.91	2.45–32.46	
No statement	62 (61%)	39 (39%)	4.40	1.23–15.75	
Party					
CDU/CSU					
Negative	4 (57%)	3 (43%)	1.00	Reference	0.416*
Positive	44 (39%)	69 (61%)	2.09	0.45–9.79	
No statement	38 (35%)	72 (66%)	2.53	0.54–11.88	
Linke					
Negative	10 (67%)	5 (33%)	1.00	Reference	0.099*
Positive	3 (30%)	7 (70%)	4.67	0.83–26.24	
No statement	25 (68%)	12 (32%)	0.96	0.27–3.44	
FDP					
Negative	4 (100%)	0 (0%)	—	—	0.688*
Positive	15 (75%)	5 (25%)	1.41	0.41–4.89	
No statement	38 (81%)	9 (19%)	1.00	Reference	
Green Party					
Negative	23 (92%)	2 (8%)	1.00	Reference	0.334*
Positive	9 (75%)	3 (25%)	3.83	0.55–26.89	
No statement	25 (89%)	3 (11%)	1.38	0.21–9.01	
SPD					
Negative	2 (100%)	0 (0%)	—	—	0.102*
Positive	19 (29%)	46 (71%)	1.32	0.64–2.71	
No statement	25 (35%)	46 (66%)	1.00	Reference	
AfD					
Negative	1 (100%)	0 (0%)	—	—	0.912*
Positive	2 (100%)	0 (0%)	—	—	
No statement	81 (94%)	5 (6%)	—	—	
Educational background					
Vocational training					
Negative	12 (86%)	2 (14%)	1.00	Reference	**<0.001** *
Positive	16 (33%)	33 (67%)	12.38	2.47–62.01	
No statement	51 (54%)	44 (46%)	5.18	1.10–24.40	
No vocational training					
Negative	32 (80%)	8 (20%)	1.00	Reference	**<0.001** *
Positive	76 (44%)	98 (56%)	5.16	2.25–11.83	
No statement	181 (64%)	102 (36%)	2.25	1.00–5.08	
Higher education degree					
Negative	36 (82%)	8 (18%)	1.00	Reference	**<0.001** *
Positive	77 (41%)	112 (59%)	6.55	2.89–14.85	
No statement	193 (62%)	121 (39%)	2.82	1.27–6.27	
No higher educational degree					
Negative	8 (80%)	2 (20%)	1.00	Reference	0.097*
Positive	15 (44%)	19 (56%)	5.07	0.93–27.48	
No statement	39 (61%)	25 (39%)	2.56	0.50–13.07	
UAS degree					
Negative	5 (83%)	1 (17%)	1.00	Reference	0.213*
Positive	6 (43%)	8 (57%)	6.67	0.61–73.03	
No statement	27 (63%)	16 (37%)	2.96	0.31–27.67	
University degree					
Negative	31 (82%)	7 (18%)	1.00	Reference	**<0.001** *
Positive	71 (41%)	104 (59%)	6.49	2.71–15.55	
No statement	166 (61%)	105 (39%)	2.80	1.19–6.59	

Significant *p*-values are highlighted in bold.

CI, coincidence interval; UAS, University of Applied Sciences; STEM, science, technology, engineering and technology.

* Fisher's Exact test.

## Discussion

Recent research suggests that there is no significant difference between opt-in and opt-out organ donation systems when implemented in isolation. Successful organ donation policies rely on clear communication, strong government support, and responsiveness to public sentiment. Hospitals need well-trained staff to identify donors and engage families in difficult conversations, alongside technical support for donor registers and waiting lists. Transparency and accountability through diligent data reporting are essential to maintaining public trust in the transplant system. What is crucial, is the development of a robust system that includes strong ethical frameworks, informed consent, and fosters trust and transparency (8).

For instance, the success of Spain's transplantation system can be attributed to three key factors: a well-established legislative framework, effective clinical leadership, and a highly organized logistics network managed by the National Transplant Organization (ONT). This approach led to a doubling of deceased organ donations in less than a decade. Additionally, strong sociopolitical support played a vital role in sustaining this success. Spain's model may offer valuable lessons for other countries aiming to improve their organ donation systems (9).

To date, trust in the German transplant system remains low, largely due to the fact that key individuals involved in the German transplantation scandal were not legally prosecuted. Guidelines for organ allocation were bypassed by falsifying urgency criteria, including faked dialysis indications, undocumented HCC status, ignored alcohol use disorder guidelines, faking data in heart and lung allocation as well as applying medications without indication. Key figures in the allocation scandal, such as the President of Eurotransplant (Bruno Meiser) being the Director of the Transplant Center in München Großhadern, member of the Permanent Committee for Organ Donation at the German Medical Association and Member of the attached Audit Committee for Transplant Centers, held influential roles in transplant commissions and Eurotransplant before 2012, when many manipulations occurred and were facilitated by changes in guidelines that allowed outpatient clinics to manage waitlist patients, thus opening the door to data manipulation in lung and heart transplantation. Despite their authority, these officials failed to push for reforms or oversee their centers, raising concerns about their inaction and accountability (10–13).

On a professional level, efforts to reform the system have focused on involving intensive care physicians and anesthesiologists more actively in the organ donation process. Politically, there has been a push to transition to an opt-out system. While sociopolitical support for this change exists among the general population, it has been lacking in political representation.

By January 2019, 57% of German MPs had not publicly expressed an opinion on organ donation. The Green Party opposed mandatory decisions on the matter and criticized the proposal to store organ donation declarations on health cards (14). Critics among MPs called for greater transparency, respect for self-determination, and increased oversight in the organ donation process (7).

Supporters of the opt-out system argued that it would help normalize organ donation, often pointing to Spain's successful model. However, opponents, particularly from the FDP, argued that silence should not be interpreted as consent. A representative from Die Linke raised concerns that vulnerable groups, such as the homeless and mentally ill, might lack the necessary information or means to formally object. Another MP from the Greens emphasized that individuals do not belong to the state. Additionally, an SPD representative warned that, under the opt-out system, relatives would lose their right to refuse, relegating them to the role of passive witnesses (4).

However, these claims are inaccurate. The draft law explicitly states that relatives can exercise the right to object on behalf of the individual. Furthermore, organ removal would not be permitted for individuals who are unable to understand the nature, significance, and implications of organ or tissue donation and align their will accordingly (15).

A majority of the German population supports organ donation. According to annual surveys by the Federal Center for Health Education, around 80% of respondents in 2022 had a positive attitude toward organ donation, and 44% possessed an organ donor card (16). Current surveys of the German population clearly demonstrate that a majority (71%) favors an opt-system as well (17). Similarly, our study found that 81% of MPs who expressed an opinion were in favor of organ donation; however, only 59% supported the opt-out system. This indicates that while many hold positive views about organ donation, this does not necessarily translate into support for the opt-out system.

In the same survey, the main reasons for opposing organ donation included a general rejection of life-prolonging measures, religious beliefs, distrust in the system, and fears of potential abuse.

The decision on the opt-out system, presented as a matter of conscience with party discipline suspended, may have been strategically influenced. Despite a generally positive attitude toward organ donation, many MPs voted against the opt-out system, indicating notable party-dependent voting patterns. This raises the question of whether MPs are truly representing the views of their constituents or if they are acting based on personal convictions or party discipline.

A positive development is the increased focus on organ donation by the German Society for Anesthesiology and Intensive Care Medicine (DGAI) and the German Interdisciplinary Association for Intensive and Emergency Medicine (DIVI). Since 2024, intensivists and anesthetists have had access to additional qualifications and a credentialing system in transplant medicine (10), highlighting their pivotal role in the donation and transplantation process. This approach mirrors the path taken by Spain over 30 years ago.

Additionally, AB0-incompatible kidney transplants typically lead to significantly poorer outcomes. Integrating these cases into cross-over living kidney transplantation programs could improve medical outcomes and reduce costs, making it a key topic for future political discussions (18).

In conclusion, the survey identified two main factors influencing voting behavior. First, there was significant opposition to the opt-out system for organ donation, with framing and whataboutism closely tied to party affiliation, particularly among members of the Green Party, AfD, and FDP. Second, many MPs demonstrated a lack of in-depth knowledge regarding transplant legislation, especially § 4, which governs that the next relatives have to be asked for the presumed consent of the deceased. Notably, despite the Green Party's firm stance against voting alongside the AfD (Firewall or “Brandmauer”), they aligned in opposing the opt-out organ donation system (19). Moreover, it highlights that in a representative democracy, MPs might consider that their vote should represent the will of their electorate.

On the other hand, a recent survey indicated that a majority of citizens is in favor for change to the opt-out system (17). This highlights the importance of fact-based discussions between political representatives and the public, which should be actively promoted by medical professionals.

## Data Availability

The raw data supporting the conclusions of this article will be made available by the authors, without undue reservation.

## References

[1] Eurotransplant. Kennzahlen Deutschland (2024). Available online at: https://www.eurotransplant.org/region/deutschland/ (Accessed October 3, 2024).

[2] Zecher D Tieken I Wadewitz J Zeman F Rahmel A Banas B. Regional differences in waiting times for kidney transplantation in Germany. Dtsch Arztebl Int. (2023) 120(23):393–9. 10.3238/arztebl.m2023.0098 37097064 PMC10433364

[3] Deutsche Stiftung Organtransplantation. Statistiken zur Organspende im Überblick: DSO (2024). Available online at: https://www.dso.de/organspende/statistiken-berichte/organspende (Accessed September 15, 2024).

[4] Deutscher Bundestag. Organspenden: Mehrheit für die Entscheidungslösung (2020). Available online at: https://www.bundestag.de/dokumente/textarchiv/2020/kw03-de-transplantationsgesetz-674682 (Accessed June 10, 2024).

[5] Bundesgesetzbuch. Gesetz über die Spende, Entnahme und Übertragung von Organen und Geweben (Transplantationsgesetz - TPG): Bundesministeriums der Justiz (1997). Available online at: https://www.gesetze-im-internet.de/tpg/TPG.pdf (Accessed June 10, 2024).

[6] Englbrecht JS Schrader D Kraus H Schafer M Schedler D Bach F How large is the potential of brain dead donors and what prevents utilization? A multicenter retrospective analysis at seven university hospitals in north Rhine-Westphalia. Transpl Int. (2023) 36:11186. 10.3389/ti.2023.11186 37252613 PMC10211426

[7] Deutscher Bundestag Drucksache 17/12225. Transparenz und öffentliche Kontrolle im Prozess der Organspende herstellen (2013).

[8] Etheredge HR. Assessing global organ donation policies: opt-in vs opt-out. Risk Manag Healthc Policy. (2021) 14:1985–98. 10.2147/RMHP.S270234 34012308 PMC8128443

[9] The Lancet. Organ donation: lessons from the Spanish model. Lancet. (2024) 404(10459):1171. 10.1016/S0140-6736(24)02128-7 39341631

[10] Nashan B Settmacher U Koch M. The German transplant certification. Hepatobiliary Surg Nutr. (2024) 13(2):382–6. 10.21037/hbsn-24-36 38617472 PMC11007333

[11] Nashan B Hugo C Strassburg CP Arbogast H Rahmel A Lilie H. Transplantation in Germany. Transplantation. (2017) 101(2):213–8. 10.1097/TP.0000000000001554 28118313

[12] Nashan B Hugo C Strassburg C Arbogast H Rahmel A Lilie H. The authors’ reply. Transplantation. (2018) 102(2):e83–4. 10.1097/TP.0000000000001987 29084023

[13] Verrel T. Absolution von Richtlinienverstößen durch Sachverständigengutachten. Medizinrecht. (2017) 35(8):597–601. 10.1007/s00350-017-4675-0

[14] Deutscher Bundestag Drucksache 17/9776. Änderungsantrag zum Entwurf eines Gesetzes zur Regelung der Entscheidungslösung im Transplantationsgesetz (2012).

[15] Bundestag D. Entwurf eines Gesetzes zur Regelung der doppelten Widerspruchslösung im Transplantationsgesetz, Drucksache 19/11096. Berlin2019.

[16] Bundeszentrale für gesundheitliche Aufklärung. Wissen, Einstellung und Verhalten der Allgemeinbevölkerung (14 bis 75 Jahre) zur Organ- und Gewebespende: BZgA (2022). Available online at: https://www.bzga.de/fileadmin/user_upload/PDF/pressemitteilungen/daten_und_fakten/BZgA_Infoblatt_Studie_Organspende_2022_final.pdf (Accessed July 15, 2024).

[17] NDR. Umfrage: Deutliche Mehrheit für Widerspruchslösung bei Organspenden: NDR (2024). Available online at: https://www.ndr.de/ndrfragt/Umfrage-Deutliche-Mehrheit-fuer-Widerspruchsloesung-bei-Organspenden,organspende826.html (Accessed October 02, 2024).

[18] Liefeldt L Glander P Friedersdorff F. [Outcome of ABO-incompatible living-donor kidney transplants: a plea for crossover living-donor kidney transplantation]. Urologie. (2024) 63(4):357–60. 10.1007/s00120-024-02316-4 38507087

[19] Gruene. Unser Programm ist beschlossen. Keine Zusammenarbeit mit der AfD (2025). Available online at: https://www.gruene.de/artikel/unser-programm-ist-beschlossen (Accessed September 4, 2024).

